# Give Me a Chance! Sense of Opportunity Inequality Affects Brain Responses to Outcome Evaluation in a Social Competitive Context: An Event-Related Potential Study

**DOI:** 10.3389/fnhum.2018.00135

**Published:** 2018-04-06

**Authors:** Changquan Long, Qian Sun, Shiwei Jia, Peng Li, Antao Chen

**Affiliations:** ^1^Key Laboratory of Cognition and Personality of the Ministry of Education, Southwest University, Chongqing, China; ^2^School of Psychology, Shandong Normal University, Jinan, China; ^3^College of Psychology and Sociology, Shenzhen University, Shenzhen, China

**Keywords:** opportunity equality, outcome evaluation, reward positivity, P3, event-related potentials

## Abstract

People are strongly motivated to pursue social equality during social interactions. Previous studies have shown that outcome equality influences the neural activities of monetary feedback processing in socioeconomic games; however, it remains unclear whether perception of opportunity equality affects outcome evaluation even when outcomes are maintained equal. The current study investigated the electrophysiological activities of outcome evaluation in different instructed opportunity equality conditions with event-related potentials (ERPs). Participants were asked to play a competitive dice game against an opponent to win money. Opportunity equality was manipulated in three conditions, depending on whether participants were allowed the opportunity to throw less, equal, or more dice compared to their opponents. Although participants received a winning outcome with approximately 50% chance in all equality conditions, they selectively exhibited sensitivity to the less-dice condition by reporting stronger feelings of unfairness and unpleasantness than in the equal and more-dice conditions. In line with the behavioral results, larger reward positivity amplitudes were elicited by the monetary outcome in the less-dice condition than in the other two conditions, reflecting intensified reward prediction error (RPE) signals under negative emotional arousal. Further, P3 amplitudes were enhanced following reward feedback only in the unequal conditions, perhaps due to the high-level motivational and affective processing associated with resolving conflict between social norms and self-interest. The present findings elucidate the complex temporal course of outcome evaluation processes in different opportunity equality conditions.

## Introduction

People show strong motivation to pursue social equality during social interactions (Hsu et al., 2008; Schäfer et al., 2015). Numerous studies have found that people usually rejected inequality offers in economic games due to inequality aversion (Fehr and Schmidt, 1999; Brosnan and de Waal, 2014). For example, previous studies have commonly adopted the ultimatum game to manipulate outcome equality in social contexts (for review see Aoki et al., 2015). In a typical ultimatum game, the proposer is required to make an offer on how to allocate a certain amount of resources, and the responder is required to decide whether to accept the offer or not. If the responder accepts the offer, the two persons share the resources as proposed; otherwise, the game ends and both players receive no reward (Güth et al., 1982). According to classical economic theories, the responders are supposed to accept any amount of resources allocated; however, people frequently refuse unequal offers to punish others if they believe that they have received an inequitable offer that favors the peer, with the aim to accomplish a more equitable outcome in the future (Fehr and Gächter, 2002; Boksem and De Cremer, 2010).

Social equality involves both outcome and opportunity equality (Lefranc et al., 2009). Opportunity equality concerns whether two or more persons receive equivalent sets of chances (Arneson, 1989; Kranich, 1996; Aoki et al., 2015) and is stressed in daily life, such as in healthcare, welfare and education (Roemer, 2002; Welzel and Inglehart, 2010; Ben-Shahar, 2016). In modern developed society, opportunity equality may be more clearly and universally valued than outcome equality (Marshall et al., 1999). Moreover, as described above, most studies have used the ultimatum game or modified ultimatum game to investigate social equality. During these games, outcome evaluation is always under an opportunity inequality situation, because the proposer has an opportunity to make an offer, whereas the responder is not afforded this opportunity (Aoki et al., 2015). To wit, opportunity equality was not systematically investigated in these studies, leaving an open question whether opportunity inequality aversion depends on the unequal and disadvantageous outcome or not. Therefore, the first aim of the present study was to test whether opportunity inequality could arouse inequality aversion and influence brain responses to outcome evaluation even when the outcome was controlled to be equal.

Notably, previous studies on social equality have exclusively focused on outcome equality (Aoki et al., 2014; for review, see Aoki et al., 2015). For example, previous event-related potential (ERP) studies have consistently found that the inequality proposal in the ultimatum or modified ultimatum game elicited a greater frontal-central negative deflection occurring at approximately 200–400 ms (Massi and Luhmann, 2015; Kaltwasser et al., 2016; Mothes et al., 2016; Wang et al., 2016). This ERP component is known as feedback-related negativity (FRN, e.g., Miltner et al., 1997; Gehring and Willoughby, 2002; Nieuwenhuis et al., 2004; Yeung et al., 2005; Hajcak et al., 2006, 2007). While a larger FRN component was originally linked to negative feedback, it has been recently proposed that the different waves between positive and negative feedback, termed Reward Positivity (RewP), were actually driven by positive feedback (Holroyd et al., 2008; Proudfit, 2015). RewP indexed a reward prediction error (RPE) signal, corresponding to the difference between the actual and the expected outcomes (Holroyd and Coles, 2002; Ferdinand et al., 2012; Sambrook and GoLin, 2015; Heydari and Holroyd, 2016). Therefore, greater amplitudes of RewP were elicited by unequal outcomes in ultimatum games, reflecting the violation of outcome equality expectancy (Mussel et al., 2014; Osinsky et al., 2014). In addition to the cognitive aspect, previous studies have also shown that short-term emotional experiences could modulate the RewP amplitudes (Hajcak et al., 2003; Bress et al., 2015; Kaltwasser et al., 2016); that is, more negative experiences intensify the RewP effect.

In addition to RewP, P3 has also been frequently observed in outcome evaluation studies (Chen et al., 2013; Liu et al., 2017). P3 refers to a positive deflection within a 300–700-ms time window following RewP (Kreussel et al., 2012; Martin, 2012). Compared to RewP, P3 has been associated to high level motivational and affective evaluation (Nieuwenhuis et al., 2004; Hajcak et al., 2010). Specifically, P3 amplitudes were modulated by several context-related factors, such as social comparison and the magnitude of the outcome during outcome evaluation (Yeung and Sanfey, 2004; Wu and Zhou, 2009; Wu et al., 2012). Additionally, the P3 component has also been found to be related to the aversion effect during evaluation of unequal outcome (Chen et al., 2013; Liu et al., 2017).

So far, very few studies have focused on brain activity involved in opportunity equality. In a recent functional magnetic resonance imaging (fMRI) study, Aoki et al. (2014) found that two important regions, the ventromedial prefrontal cortex (vmPFC) and ventral striatum (VS), were selectively recruited by opportunity valuation processing and reward outcome processing in an indirectly competitive game. During the opportunity stimuli stage, the activity of the vmPFC was sensitive to opportunity equality. During the reward stimuli stage, the activity of both the vmPFC and VS was sensitive to self-reward and the activity of the vmPFC was sensitive to outcome equality. Although interpersonal opportunity equality and personal reward information were spatially distinguished in this study, it remains unknown how participants processed these two types of information temporally. Thus, the present study also aimed to explore the temporal dynamics of opportunity equality during outcome evaluations by using ERPs because the ERP analysis of cognitive processes is known to have superior temporal resolution (Luck, 2014).

In this study, participants joined a dice game to compare their dice score to that of an opponent’s. Three opportunity conditions were manipulated: (1) less-dice (lower number of dice than their opponents); (2) equal-dice (same number of dice as their opponents); and (3) more-dice (larger number of dice than their opponents). The outcomes depended on the two dice scores, yielding two outcomes for participants: win (larger score than their opponents) and loss (lower score than their opponents). Importantly, the probability of monetary winning was kept the same in these three opportunity conditions for participants. Our previous study found that different instructions dramatically influenced participant behavior and electrophysiological activity in a gambling task, even when the remaining experimental conditions remained identical (Li et al., 2011). Here, our manipulation of opportunity equality was also mainly based on the instruction and “cover story” presented to the participants.

Strong feelings of unfairness during opportunity inequality emerge when there are restrictions in the probability to obtain better outcomes or to control the environment and to experience ourselves as active beings (Fujiwara et al., 2013; Aoki et al., 2015). Therefore, we expected that less-dice for participants would induce stronger feelings of unfairness and unpleasantness than would equal-dice and more-dice. Given that the RewP component reflects a rapid evaluation of outcome based on self-interest preference (Koban et al., 2012; Rak et al., 2013) and could be influenced by short-term negative emotion (Hajcak et al., 2003; Bress et al., 2015; Kaltwasser et al., 2016), we predicted that larger RewP amplitudes would be observed in the less-dice condition than under the other conditions because of the subjective increased inequality aversion in this disadvantageous context. The P3 component has been shown to reflect high-level of motivational significance during outcome evaluation, such as inequality aversion (Hajcak and Olvet, 2008; Hajcak et al., 2010; Martin, 2012). Thus, we also hypothesized that P3 could reflect the interaction effect between outcome and opportunity equality beyond self-interest motivation.

## Materials and Methods

### Participants

Twenty-six healthy undergraduates (15 women; mean age: 19.57 years, *SE*: 0.31, range from 18 to 23 years) participated in the experiment. All participants were right-handed and had normal or corrected-to-normal eyesight. The present study was approved by the ethics review board at Southwest University’s Faculty of Psychology. All subjects provided written informed consent in accordance with the Declaration of Helsinki. During the post-experiment debriefing, all the participants reported that they believed that they had played the game against a real person.

### Design, Materials and Procedure

A two-person dice game was used. Participants had one, two, or three dices to roll in a trial and the opponents had two dices in all trials. Thus, three opportunity conditions were presented: less-dice (1 for participants vs. 2 for their opponents, less), equal-dice (2 for participants vs. 2 for their opponents, equal), and more-dice (3 for participants vs. 2 for their opponents, more). Outcomes involved win and loss for the participants. Therefore, this study involved six sub-conditions: win during less-dice (less-win), loss during less-dice (less-loss), win during equal-dice (equal-win), loss during equal-dice (equal-loss), win during more-dice (more-win), and loss during more-dice (more-loss). Each sub-condition was repeated 48 times. Therefore, 288 trials were involved in total. Trials were presented randomly. After every 36 trials, participants were allowed to rest.

Before the experiment, each participant was introduced to a same-sex opponent, who was actually a lab assistant. Participants were informed that they would play a dice game with the opponent in a different room but connected by computers. Afterward, participants sat in front of the computer screen at a distance of 1 m and were instructed that they may have one, two, or three dices in each trial and the opponents would always have two dices in every trial. The hands were presented on the screen. The downward-pointing hand represented participants, and the upward-pointing hand represented their opponents. Similar to Li et al. (2010), to increase self-engagement, participants were asked to press the “G” key to stop a yellow moving bar for tossing dices. The participant was required to press the “G” key once for each dice they had at their disposal. Moreover, participants were informed that the longer the yellow bar was, the greater the strength of tossing dice would be.

Participants were informed that each dice could produce one of six outcomes, 1, 2, 3, 4, 5 and 6. The score in each trial was defined by the maximum dice score produced by any of the available dice. For example, if a participant had only one dice in a trial and the dice score was 5, then the score of the participant in this trial was 5. Accordingly, if a participant had two dice in a trial and the dice scores were 1 and 3, then the score of the participant in this trial was 3. The outcome of this game was determined by comparing the scores of the participant and the opponent. If the participant’s score was higher than the opponent’s in a trial, the participant would gain 0.5 yuan and the opponent would lose 0.5 yuan. Otherwise, the participant would lose 0.5 yuan and the opponents would gain 0.5 yuan. Participants were informed that they started with 30 yuan as a basic reward, and the final reward would be related to their performance. In fact, the game was controlled by a computer program, with a 50:50 win:lose ratio under each opportunity condition. In order to increase participant engagement in the task, our participants were notified that their final reward consisted of two parts. The first part was the basic reward (30 Yuan) plus the monetary winnings in each trial in the ERP experiment. The second part would be calculated after the end of the experiment based on their relative performance compared with that of other participants. As the chance of winning was 50:50 across conditions, no participant would actually receive an extra reward. After the ERP experiment, all participants were informed that they “did a relatively good job” and received 15 extra Yuan for overall performance. Therefore, the final reward for each participant was predetermined at 45 Yuan.

**Figure 1:**
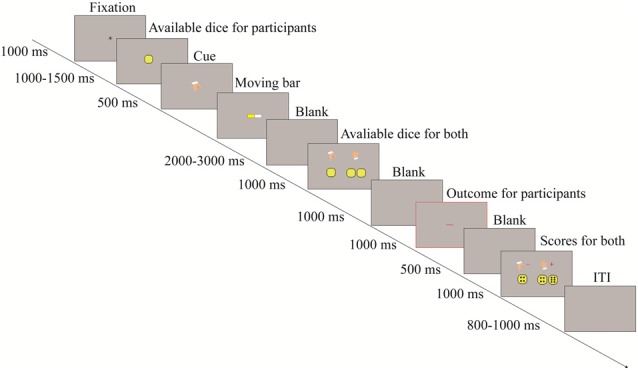
The procedure of the two-person dice game.

Figure 1 shows the procedure followed in this study. In each trial, a “*” was presented at the center of the screen. Then, the numbers of the yellow circles displayed to represent the numbers of available dice for the participants. That is, one, two, or three yellow circles denoted that the participants had one, two, or three dice, respectively. After the circles, a downward pointing hand was presented to cue participants to prepare to toss the dice. After the cue, there was a moving yellow bar, which required participants to press the “G” key to stop the bar for tossing the dice. After pressing the key, a blank screen was displayed for a random interval of 2000–3000 ms. After the blank screen, the available dice of each person were presented without showing dice scores, only to suggest that both persons had completed the toss. Then, another blank screen was displayed. Next, the outcome for the participants was displayed with a red “+” or a red “−”. The red “+” denoted gain and the red “−” denoted loss. The outcome had a visual angle of 2.2° × 1.6° (5.52 cm × 2.38 cm, width × height). Finally, all dice scores were shown. After a blank screen displayed for a random interval between 800 ms and 1000 ms, the next trial began. Before the formal experiments, participants performed 12 practice trials to understand the task and the key operation. These practice trials were excluded from further analysis.

When all trials had completed, participants were instructed to assess the degree of fairness and pleasantness for each opportunity condition, using a seven-point Likert scale (1 = extremely unfair, 7 = extremely fair or 1 = extremely unpleasant, 7 = extremely pleasant). For example, participants were asked to judge how fair they felt it was when they had one dice to roll and the opponent had two or to judge how pleasantly they felt when they had two dice to roll and the opponent also had two.

### EEG Data Acquisition and Pre-processing

The electroencephalogram (EEG) was recorded while the participants were performing the trials. Sixty-four scalp electrodes were placed on a Neuroscan cap (Neuroscan, Herndon, VA, USA), according to the criteria of the international 10–20 system. The vertical electrooculogram (EOG) was recorded supra- and infra-orbitally at the left eye; the horizontal EOG was recorded from the left vs. the right orbital rim. The EEG and EOG were amplified with the SynAmps2 amplifier (Neuroscan, Herndon, VA, USA) and digitized at the 500 Hz channel. Electrode impedances were maintained below 5 KΩ throughout the experiment.

The off-line data were analyzed using the EEGLAB toolbox (EEGLAB v12.0.2.4b, Delorme and Makeig, 2004) and the ERPLAB toolbox (ERPLAB v6.0, Lopez-Calderon and Luck, 2014) under Matlab R2012a (MathWorks, Natick, MA, USA). Only the EEGs elicited by the outcome in the participants were analyzed. The offline filtered passband was at the half-power cutoff between 0.1 Hz and 30 Hz, with an IIR-Butterworth filter (roll-off = 12 dB/oct). Artifacts (blinks, eye-movements and muscle-movements) were corrected by independent component analysis. The data were referenced with the averaged amplitudes of the left and right mastoids (Luck, 2014). The averaged epoch was 1000 ms, including a 200 ms pre-feedback baseline. Trials from the six sub-conditions (less-win, less-loss, equal-win, equal-loss, more-win, more-loss) were selected and averaged separately. Trials with absolute EEG voltages exceeding ± 75 μV were excluded from averaging. Finally, there were 46.65 trials (*SE*: 1.60) for less-win, 46.65 trials (*SE*: 0.47) for less-loss, 46.42 trials (*SE*: 0.33) for equal-win, 46.46 trials (*SE*: 0.38) for equal-loss, 46.46 trails (*SE*: 0.44) for more-win, and 46.81 trials (*SE*: 0.27) for more-loss.

### Data Analysis

For the subjective assessments, both the fairness and the pleasantness ratings were analyzed. One-way repeated measures analysis of variance (ANOVA) was conducted for the fairness and the pleasantness ratings separately, with the opportunity conditions (less, equal and more) as repeated factors.

**Figure 2:**
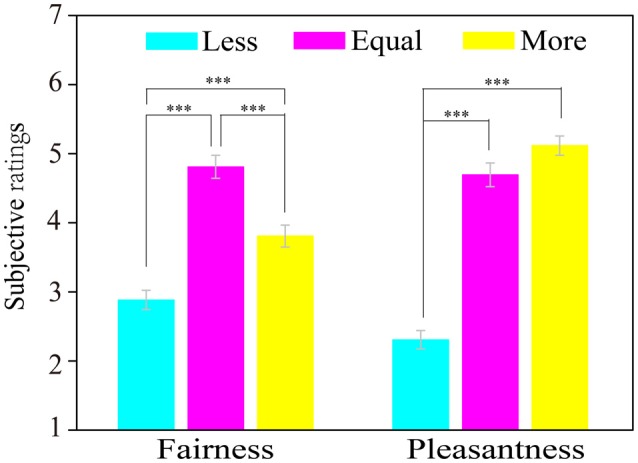
Result of subjective assessments: fairness and pleasantness. Error bars represent standard errors. ***p < 0.001.

For the RewP analysis, based on a meta-analysis of RewP studies by Sambrook and GoLin (2015), a loss-win difference wave was assessed (see Figure 2) by subtracting the win from the loss in each opportunity condition, and the mean RewP amplitudes were measured in the 250–300-ms time window at FCz. This time window and electrode position were selected because previous studies have shown that the RewP effect typically reaches its maximum amplitude at this site and in this time window (Yeung et al., 2005; Holroyd et al., 2008; Heydari and Holroyd, 2016). One-sample *t*-tests were conducted on the RewP amplitude in each condition against the baseline (0 μV) to test whether there was a robust RewP effect in each condition. Further, one-way repeated-measures ANOVA was used to analyze the RewP effect under the three opportunity conditions, with the opportunity conditions (less, equal and more) as repeated factors.

Previous studies have found that P3 amplitudes are sensitive to various psychological processes and usually peak at the midline electrodes on the scalp (e.g., Delplanque et al., 2004; Li et al., 2010; Qiu et al., 2010). To explore the P3 effect in the present novel paradigm, the mean amplitudes of P3 were measured in the 300–600-ms time window at Fz, FCz, Cz, CPz and Pz. Three-way repeated-measures ANOVA was applied, with opportunity conditions (less, equal and more), outcomes (win and loss), and electrodes (Fz, FCz, Cz, CPz and Pz) as independent factors. For all analyses, the degrees of freedom of the F ratio were corrected for deviations according to the Greenhouse-Geisser method when the model did not satisfy the sphericity assumption. *Post hoc* testing of significant effects was corrected using the Bonferroni method.

Correlation analyses were performed for each opportunity condition to illustrate the potential relation between the EEG data (RewP and P3) and the subjective ratings (the feeling of unfairness and unpleasantness). The P3 difference amplitudes (loss subtract win) at Cz were used in the correlation analyses because of the observed P3 amplitudes peaking at Cz in this study. First, the potential association between RewP and the subjective ratings was analyzed using two-tailed Spearman rank correlation analysis. In addition, partial correlation analyses were conducted to exclude the effect of P3 difference amplitudes. Similar to RewP, the potential association between P3 and the subjective ratings was also analyzed using two-tailed Spearman correlation analysis; partial correlation analyses were conducted to exclude the influence of RewP amplitudes.

## Results

### Behavioral Results

Figure 2 shows the averaged fairness and pleasantness ratings. For the fairness ratings, the data revealed a significant main effect of opportunity conditions, *F*_(2,50)_ = 69.79, *p* < 0.001, $\eta_{\text{p}}^{\text{2}}$ = 0.74. *Post hoc* analysis found that the fairness rating scores for equal-dice (*M* = 4.81, *SE* = 0.17) were significantly higher than for less-dice (*M* = 2.88, *SE* = 0.14; *p* < 0.001) and for more-dice (*M* = 3.81, *SE* = 0.16; *p* < 0.001), and the fairness rating scores for more were significantly higher than for less-dice (*p* < 0.001). Thus, the feeling of fairness was highest under the equal-dice condition, and the feeling of fairness was higher under the more-dice than under the less-dice condition.

For the pleasantness ratings, the data revealed a significant main effect of opportunity conditions, *F*_(2,50)_ = 142.90, *p* < 0.001, $\eta_{\text{p}}^{\text{2}}$ = 0.85. *Post hoc* analysis suggested that the pleasantness ratings for equal-dice (*M* = 4.69, *SE* = 0.17) and more-dice (*M* = 5.12, *SE* = 0.14) were significantly higher than for less-dice (*M* = 2.31, *SE* = 0.13; both *p* < 0.001), whereas the difference in the pleasantness ratings for more-dice were not significantly higher than for equal-dice (*p* = 0.16). Thus, participants felt more unpleasant under the less-dice condition than under the equal-dice and more-equal conditions.

### ERP Results

**Figure 3:**
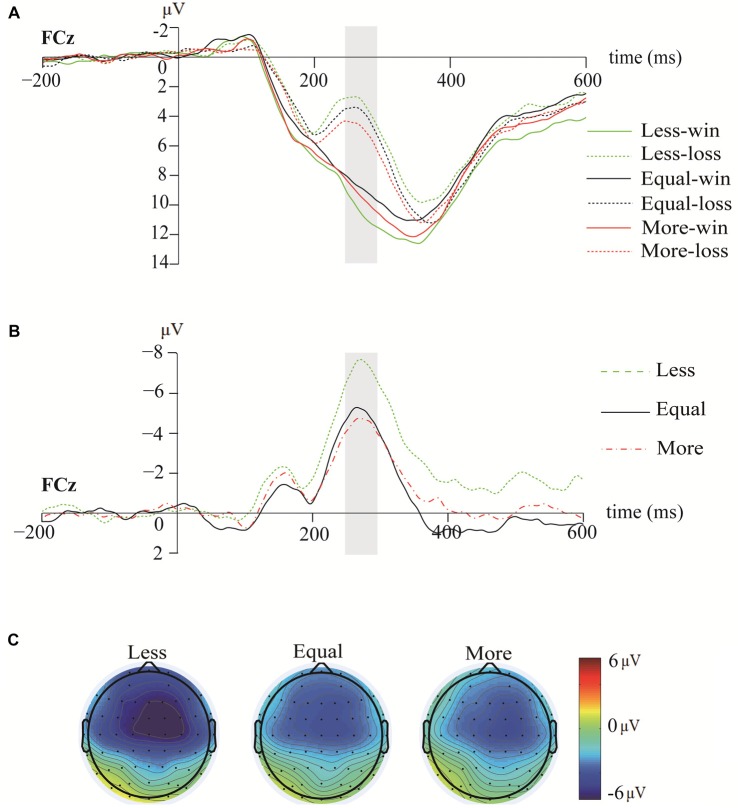
The grand-average event-related potential (ERP) waveforms, reward positivity (RewP) waveforms and topographies. **(A)** The grand-average ERP waveforms at FCz. **(B)** The RewP waveforms elicited by less-dice, equal-dice and more-dice at FCz. **(C)** The scalp distributions of RewP at the 250–300-ms time window among the three opportunity conditions.

**Figure 4:**
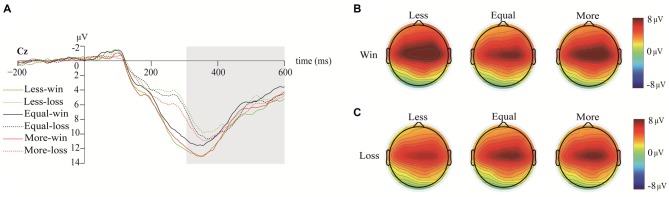
The grand-average ERP waveforms and topography among opportunity conditions under win and loss. **(A)** The P3 waveforms at Cz among opportunity conditions under both win and loss outcomes. **(B)** The topographies of three opportunity conditions at 300–600 ms under win. **(C)** The topographies of three opportunity conditions s at 300–600 ms under loss.

**Figure 5:**
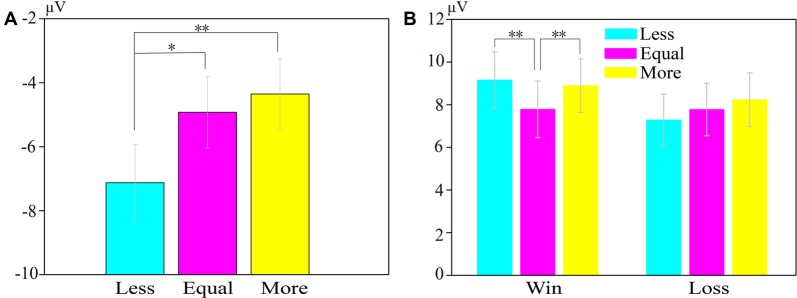
The grand-average bar chart for the amplitudes of RewP and P3. **(A)** The RewP amplitudes elicited by less-dice, equal-dice and more-dice at FCz. **(B)** The P3 amplitudes elicited by less-dice, equal-dice and more-dice at Cz. Error bars represent standard errors. *p < 0.05, **p < 0.01.

Figure 3 illustrates the grand-averaged ERP waveforms at FCz, the wave difference between loss and win (loss subtract win) at FCz, and the topographies of RewPs in the 250–300-ms time window. Figure 4 illustrates the grand-averaged ERP waveforms at Cz and the topographies of P3 for each opportunity condition under both win and loss at the 300–600-ms time window. The bar charts in Figure 5 illustrate the mean amplitudes of RewP and P3 in each condition.

### RewP

The results showed that the RewP amplitudes were significantly different from baseline (0 μV) in each opportunity condition (less: *t*_(25)_ = −5.94, *p* < 0.001, Cohen’s *d* = 2.37; equal: *t*_(25)_ = −4.40, *p* < 0.001, Cohen’s *d* = 1.76; more: *t*_(25)_ = −3.95, *p* < 0.01, Cohen’s *d* = 1.58). The results also showed a significant main effect of opportunity conditions, *F*_(2,50)_ = 8.72, *p* < 0.01, $\eta_{\text{p}}^{\text{2}}$ = 0.26. *Post hoc* analysis showed that the RewP amplitudes elicited by less-dice (*M* = −7.13 μV, *SE* = 1.20) were larger than those elicited by equal-dice (*M* = −4.93 μV, *SE* = 1.11; *p* < 0.05) and more-dice (*M* = −4.36 μV, *SE* = 1.10; *p* < 0.01). However, equal-dice and more-dice elicited similar RewP amplitudes (*p* = 0.99).

### P3

The results showed a significant main effect of opportunity conditions (*F*_(2,50)_ = 3.29, *p* = 0.04, $\eta_{\text{p}}^{\text{2}}$ = 0.12), outcomes (*F*_(1,25)_ = 5.20, *p* = 0.03, $\eta_{\text{p}}^{\text{2}}$ = 0.17), and electrodes (*F*_(4,100)_ = 10.58, *p* < 0.01, $\eta_{\text{p}}^{\text{2}}$ = 0.30). The results also showed a significant interaction effect between opportunity conditions and outcomes, *F*_(2,50)_ = 7.24, *p* < 0.01, $\eta_{\text{p}}^{\text{2}}$ = 0.23. The other interaction effects were not significant (all *p* > 0.05).

Further analysis for the interaction between opportunity conditions and outcomes showed that when participants won under the equal-dice condition, the elicited P3 amplitudes were lower than when they won under the less-dice and more-dice conditions (both *p* < 0.01), while the difference in P3 amplitudes between the less-dice and more-dice conditions were not significant (*p* = 0.89). In contrast, when participants lost, no significant differences were observed among the elicited P3 amplitudes in any of the three opportunity conditions, *F*_(2,50)_ = 2.28, *p* = 0.11. *Post hoc* analysis for the main effect of electrodes showed that the P3 component peaked at Cz (less-win: *M* = 9.16, *SE* = 1.32; less-loss: *M* = 7.29, *SE* = 1.21; equal-win: *M* = 7.78, *SE* = 1.33; equal-loss: *M* = 7.77, *SE* = 1.23; more-win:* M* = 8.89, *SE* = 1.25; more-loss:* M* = 8.23, *SE* = 1.26).

### The Relationship Between ERP Components and Subjective Ratings

**Figure 6:**
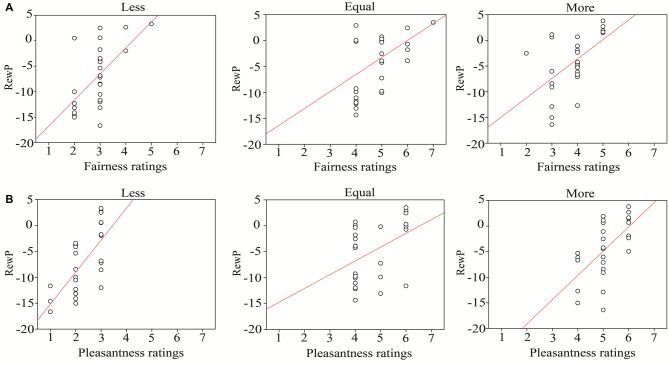
The correlations between behavioral subjective ratings and RewP amplitudes. **(A)** The correlations between fairness ratings and RewP amplitudes in each opportunity condition. **(B)** The correlations between pleasantness ratings and RewP amplitudes in each opportunity condition.

As shown in Figure 6, the results revealed a significant positive correlation between the RewP amplitudes and subjective rating data in each opportunity condition (fairness: (less: *r* = 0.60, *p* < 0.01; equal: *r* = 0.52, *p* < 0.01; more: *r* = 0.61, *p* < 0.01); pleasantness: (less: *r* = 0.69, *p* < 0.001; equal: *r* = 0.41, *p* = 0.04; more: *r* = 0.64, *p* < 0.001)). Note that the correlation remained significant when the P3 effect was controlled (fairness: (less: *r* = 0.60, *p* < 0.01; equal: *r* = 0.45, *p* = 0.02; more: *r* = 0.52, *p* < 0.01); pleasantness: (less: *r* = 0.58, *p* < 0.01; equal: *r* = 0.46, *p* = 0.02; more: *r* = 0.47, *p* = 0.02)). However, P3 amplitudes were not significantly correlated with subjective rating data in any condition, except for the fairness ratings in the equal-dice condition (*r* = 0.49, *p* = 0.010) and the pleasantness ratings in the less-dice condition (*r* = 0.41, *p* = 0.04). Notably, the correlations did not reach significance when the RewP effect was controlled (both *p* > 0.05).

## Discussion

Here, the responses to win and loss outcomes under conditions participants perceived as either fair or unfair to reveal how and when opportunity equality affected outcome evaluation were explored using ERPs. Behavioral results showed that the less-dice condition aroused stronger feelings of unfairness and unpleasantness than were elicited by the equal-dice and more-dice conditions. The ERP results showed that larger RewP amplitudes were elicited by feedback under the less-dice than did under the equal-dice and more-dice conditions. Moreover, a significant interaction between opportunity conditions and outcomes was observed on P3 amplitudes. These results suggest that opportunity equality modulated brain responses to outcome evaluation in different opportunity conditions.

Participants’ subjective ratings suggested that they were sensitive to opportunity equality. Consistent with the definition of opportunity equality (Arneson, 1989; Kranich, 1996), participants’ subjective ratings of fairness revealed that they felt a linearly decreased sense of fairness from the equal to the more-dice condition and to the less-dice condition. This result confirms that the different levels of equality were successfully manipulated by instruction and the “cover story” in our present paradigm. Moreover, participants reported more unpleasantness in the less-dice condition than in the other two conditions, as predicted. Thus, less-dice induced stronger feelings of unpleasantness than were induced by equal-dice and more-dice. These subjective rating data supported the notion that social fairness in an interpersonal context is perceived in a self-serving way (Qiu et al., 2010; Feng et al., 2013).

EEG data suggested that participants’ perception of opportunity inequality modulated their rapid outcome evaluation, as shown by the RewP amplitudes. In line with the results of previous studies, the results in this study showed a robust RewP effect in the difference wave between the win and loss feedbacks under the three opportunity conditions (Proudfit, 2015; San Martín et al., 2016). Moreover, the present results showed that less-dice elicited more negative RewP amplitudes than equal-dice and more-dice, possibly due to the general association between less-dice and disadvantageous outcomes. The possible explanation for the enhanced RewP amplitude in the less-dice condition may be the negative emotions aroused in this condition. Previous studies have found that RewP amplitudes were modulated by short-term emotional experiences (Hajcak et al., 2003; Bress et al., 2015; Kaltwasser et al., 2016). For example, Liu et al. (2017) found that negative emotional arousal increased the sensitivity to RPEs, and then elicited larger RewP amplitudes. In their study, participants joined a dictator game (a modified ultimatum game) first and then joined a gambling task. They found that larger RewP amplitudes were elicited in the gambling task when participants experienced harsh treatment (gained less than the opponents) in the dictator game than when they experienced favorable treatment (gained more than the opponents) or equal treatment (gained equal to the opponents). Liu et al. (2017) proposed that participants who received harsh treatment were in disadvantageous circumstances. The disadvantageous circumstances would arouse the participants’ negative emotions and then intensify the RPE signals to elicit larger RewP amplitudes. In addition, the results showed that the RewP amplitudes were closely related to the fairness and pleasantness feelings elicited in each condition, suggesting that the activities of the reward system covaried with the subjective experience of emotion on an individual level. Another possibility is that a larger RPE could be elicited when participants won in the less-dice condition with the lowest probability to win, which in turn elicited larger RewP amplitudes. However, we are cautious in proposing this argument because the RewP amplitude was calculated by the difference wave approach and both the unexpected winning and expected losing events could modulate the RewP amplitude in the less-opportunity condition.

The results also suggested that opportunity equality modulated the later phase of the evaluation processes, as shown by the P3 amplitudes. The P3 amplitudes were influenced by various factors during outcome evaluation, involving social comparison (Wu et al., 2011, 2012), outcome valence (Hajcak et al., 2007; Wu and Zhou, 2009), and outcome equality (Kreussel et al., 2012). We also found a significant interaction between opportunity equality and outcome; larger P3 amplitudes were elicited by the unequal (less-dice and more-dice) than by the equal condition only in reward feedback. P3 is considered to be associated with high level motivational and affective evaluation (Hajcak et al., 2010; Martin, 2012). In this study, the P3 amplitudes may reflect the affective conflict between social norms and self-interest. Despite the fact that monetary reward generally arouses positive emotion, opportunity inequality may induce negative emotional context because social norms are violated in unequal conditions, regardless of whether opportunity inequality was profitable or not. In addition, the distinctive pattern of P3 between win and loss provided additional evidence that there was an asymmetry between gains and losses in social outcome evaluation (Luo et al., 2015).

The observed experimental dissociation between the RewP and P3 effects may provide electrophysiological evidence of the two separated systems in fairness-related decision making. Previous studies suggested that fairness-related decision making involved two separated systems: System 1 and 2. System 1 involves a reflexive and intuitive proposal; System 2 involves a reflective and deliberative phase to adjust the reflexive and intuitive proposal based on social context (Feng et al., 2015; Sun et al., 2015). More specifically, System 1 was related to the initial evaluations of inequality and the negative emotional response to equality violations, noting people’s prosocial preferences (Zaki and Mitchell, 2011, 2013); System 2 was involved in detection and reconciliation of motivational conflict between social equality norm enforcement and economic self-interest, which would be conducive to further flexible decision making (Baumgartner et al., 2011; Feng et al., 2015). Accordingly, the greater RewP amplitudes observed in the less-dice condition may suggest the reflexive and intuitive proposal on opportunity equality during outcome evaluation. Moreover, the P3 amplitudes might suggest the reflective and deliberative phase in System 2 to resolve the conflict between social norms and self-interest during outcome evaluation. However, further research is required to confirm this hypothesis.

This study had some limitations. For example, we only explored the temporal dynamics of opportunity equality during outcome evaluations in a competitive situation. Further work is needed to explore the cognitive processes involved in opportunity equality during outcome evaluations in noncompetitive situations. In addition, previous studies have indicated that outcome equality is a culturally constructed behavioral norm (for review, see Schäfer et al., 2015). Future research is required to explore whether opportunity equality is also a culturally constructed behavioral norm.

## Conclusion

The present findings contribute in the elucidation of the brain activities involved in complex processes during outcome evaluation in different opportunity equality conditions. Participants were sensitive toward opportunity equality, as shown by the stronger feelings of unfairness and unpleasantness induced under the less-dice condition. The ERP results suggested that intensified RPEs under negative emotional arousal were produced, as shown by the larger RewP amplitudes elicited under the less-dice condition rather than under the other two conditions. The significant correlation results between the RewP amplitudes and the behavioral data demonstrated that these intensified RPE signals covaried across individuals. Following RewP, stronger motivational and affective evaluations, which may correspond to reflective and deliberative processes, were executed, as shown by a significant interaction effect between opportunity equality and outcome on P3 amplitudes.

## Author Contributions

CL, QS and SJ designed this experiment. CL and QS analyzed the data. CL, QS, PL and AC wrote the article.

## Conflict of Interest Statement

The authors declare that the research was conducted in the absence of any commercial or financial relationships that could be construed as a potential conflict of interest.
